# A PEGylated PVDF Antifouling Membrane Prepared by Grafting of Methoxypolyethylene Glycol Acrylate in Gama-Irradiated Homogeneous Solution

**DOI:** 10.3390/ma17040873

**Published:** 2024-02-14

**Authors:** Ting Wang, Zhengchi Hou, Haijun Yang, Jun Hu

**Affiliations:** 1 Shanghai Institute of Applied Physics, Chinese Academy of Sciences, Shanghai 201800, China; wangting@sinap.ac.cn (T.W.); hujun@sinap.ac.cn (J.H.); 2 University of Chinese Academy of Sciences, Beijing 100049, China; 3 Shanghai Synchrotron Radiation Facility (SSRF), Shanghai Advanced Research Institute, Chinese Academy of Sciences, Shanghai 201204, China; yanghj@sari.ac.cn

**Keywords:** polyvinylidene fluoride, methoxypolyethylene glycol acrylate, antifouling, simultaneous irradiation, ultrafiltration membrane

## Abstract

In this study, methoxypolyethylene glycol acrylate (mPEGA) served as a PEGylated monomer and was grafted onto polyvinylidene fluoride (PVDF) through homogeneous solution gamma irradiation. The grafting process was confirmed using several techniques, including infrared spectroscopy (FTIR), thermodynamic stability assessments, and rotational viscosity measurements. The degree of grafting (DG) was determined via the gravimetric method. By varying the monomer concentration, a range of DGs was achieved in the PVDF-g-mPEGA copolymers. Investigations into water contact angles and scanning electron microscopy (SEM) images indicated a direct correlation between increased hydrophilicity, membrane porosity, and higher DG levels in the PVDF-g-mPEGA membrane. Filtration tests demonstrated that enhanced DGs resulted in more permeable PVDF-g-mPEGA membranes, eliminating the need for pore-forming agents. Antifouling tests revealed that membranes with a lower DG maintained a high flux recovery rate, indicating that the innate properties of PVDF could be largely preserved.

## 1. Introduction

The issue of water scarcity has garnered growing attention in recent years [1]. Membrane technology, due to its energy efficiency, scalability, and consistent effluent quality, has emerged as a promising solution for ensuring a dependable water supply [2,3]. Specifically, ultrafiltration membranes, characterized by pore sizes ranging between 5 and 100 nm, demonstrate notable efficacy in eliminating viruses, proteins, and colloidal contaminants at relatively low applied pressures, setting them apart from alternative membrane technologies [4]. Consequently, ultrafiltration membranes have found extensive application in drinking water treatment facilities and urban wastewater treatment plants, capitalizing on their inherent advantages [5,6,7,8]. Polyvinylidene fluoride (PVDF), renowned for its high mechanical strength, exceptional chemical resistance, and robust thermal stability, is a popular choice in separation membrane applications [9,10,11,12]. However, its inherent hydrophobicity often leads to membrane fouling [13,14], underscoring the necessity of modifying PVDF membranes to enhance their performance.

Numerous studies have demonstrated the effectiveness of various hydrophilic materials in improving the antifouling properties of PVDF membranes [10,15,16,17,18,19,20,21,22,23]. In this context, our research group has explored grafting different monomers onto membrane materials, including N-vinylpyrrolidone (NVP) [9], methacrylic acid (MAA) [24,25,26], acrylic acid (AA) [27], and polyethylene glycol methacrylate (PEGMA) [28]. Among these, PEGylated monomers have shown superior antifouling capabilities. Previous research has established PEG materials as effective in reducing nonspecific protein adsorption [16,17]. In aqueous environments, PEG’s ability to form a hydration layer on its surface, coupled with the rapid mobility of its hydrated chains, can alter the microscopic thermodynamics at the protein–surface interface. This alteration potentially hinders the adsorption or adhesion of proteins [18].

Methoxypolyethylene glycol acrylate (mPEGA), a notable PEGylation reagent, effectively enhances the hydrophilicity and antifouling properties of materials [19,20]. Wang et al. [29] utilized UV-induced free radical polymerization to graft mPEGA onto silicone hydrogels, noting a significant decrease in the static water contact angle as the grafting degree increased. This modification reduced the adsorption of a single protein to the modified silicone hydrogel by 70–80%. Bozukova et al. [30] implemented surface modification of poly (HEMA-co-MMA) hydrogels with oligoethylene glycol methacrylate (OEGA) using atom transfer radical polymerization (ATRP) at 25 °C. This process enabled the grafting of poly(ethylene glycol) (PEG) chains onto the hydrogel surface, with findings indicating that even short poly(OEGA) brushes significantly enhanced the hydrogels’ antifouling characteristics, as verified by in vitro biotests. Similarly, Asatekin et al. [31] and Barroso et al. [32] used acrylonitrile and mPEGA as raw materials, producing PAN-g-PEO through free radical polymerization. They then prepared a PAN-based UF membrane with added graft polymer, exhibiting substantial resistance to irreversible fouling. Further, studies involving grafting or blending methoxy polyethylene glycol (mPEG) into PSf [33,34], PES [35], and PVDF [36] membranes have shown remarkable improvements in their antifouling characteristics, thereby validating the effectiveness of mPEG chain segments in enhancing these properties.

Numerous methods have been explored for PVDF PEGylation, including ozone-activated surface grafting [37] and bulk grafting [38], atom-transfer radical-polymerization (ATRP) surface grafting [39] and bulk grafting [40,41], plasma surface grafting [42], and radiation grafting [43]. While these techniques have unique characteristics, they also face specific limitations. Surface grafting can lead to irregular membrane surface structures, potentially altering pore size and distribution, which may subsequently reduce filtration performance [44,45]. In ozone-activated bulk grafting of PVDF, significant material degradation can occur [46], potentially impairing membrane properties. Moreover, the high bond energy of C-F bonds in PVDF affects both the degree and uniformity of ATRP grafting [47], and the monomer-to-PVDF concentration ratio is substantial (10:1) [41].

Gamma radiation-induced grafting, known for its superior penetrating power and mild reaction conditions, is an effective method for polymer modification. This grafting reaction is initiated by high-energy radiation, eliminating the need for an additional initiator [48,49]. Our research group’s findings indicate that gamma radiation-induced grafting yields more uniform grafting compared to heterogeneous grafting methods. However, it is noteworthy that, unlike NVP, common small molecular monomers generally cannot undergo homogeneous radiation grafting at high concentrations, leading to challenges such as insolubility and significant homopolymerization. In contrast, PEGylated monomers do not encounter these issues. Despite numerous studies demonstrating mPEGA as an effective antifouling material, reports of mPEGA-grafted modified PVDF membranes are scarce.

**Figure 1:**
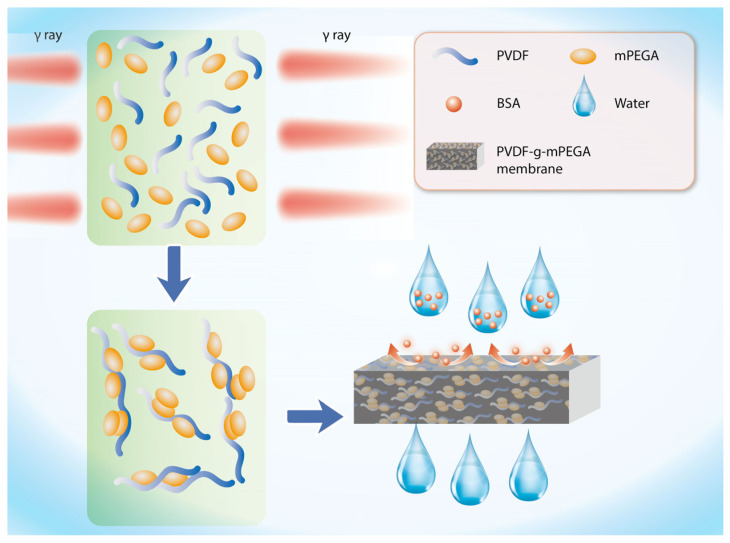
Schematic diagram of the synthesis/fabrication process and antifouling performance of membrane.

In this study, mPEGA was successfully grafted onto PVDF through homogeneous solution radiation grafting. The resultant polymer, PVDF-g-mPEGA, was then directly formed into a membrane using non-solvent-induced phase separation (NIPS). The degree of grafting (DG) was determined using the gravimetric method, and a kinetic study was conducted to investigate factors influencing DG. Additionally, the thermodynamic stability of the grafted product was analyzed by constructing a phase diagram via cloud point titration. The viscosity of the copolymer was measured using a rotational viscometer, and its molecular structure was characterized by Fourier transform infrared spectroscopy (FTIR). The hydrophilicity, surface morphology, and filtration performance of the membrane were examined using scanning electron microscopy (SEM) and water contact angle measurements. The antifouling performance of PVDF-g-mPEGA was assessed by evaluating the recovery rate of membrane filtration of bovine serum albumin (BSA) solution and determining water flux post-water wash, with comparisons drawn to the antifouling performance of PVDF-g-NVP membranes. In this research, BSA served as the model pollutant. The schematic diagram of the study is shown in Figure 1.

## 2. Materials and Methods

### 2.1. Materials

PVDF (Solef 6020, Mn = 670,000) was procured from Solvay Co. Ltd. and subjected to pre-use drying at 80 °C in a vacuum oven for 24 h. NMP, NaCl, Na_2_HPO_4_, KCl, KH_2_PO_4_, HCl, polyethylene glycols (PEG2000), and BSA were acquired from Sinopharm Chemical Reagent Co. Ltd., Beijing, China, while mPEGA (Mn = 480) was obtained from Sigma-Aldrich (Shanghai) Trading Co. Ltd., Shanghai, China. Additionally, water purified through a Milli-Q system from Millipore was employed.

### 2.2. Synthesis of Graft Copolymer

PVDF powder and N-methyl-2-pyrrolidone (NMP) were combined in an Erlenmeyer flask as per the ratios specified in Table 1. This mixture was stirred continuously at 70 °C for 24 h to achieve complete dissolution of PVDF in NMP. Once stirring was completed, the solution was allowed to cool to room temperature. Subsequently, varying amounts of mPEGA monomer were added to the flask and stirred until the solution became uniform and transparent. The mixture was then transferred to a glass tube, which was purged with nitrogen gas for 30 min before being sealed. The prepared samples were subjected to gamma-ray irradiation from a source at room temperature for a predetermined duration. Post-irradiation, the solution was carefully poured into an ethanol solution and subsequently into hot water to remove any homopolymer and unreacted monomer. Finally, the resulting precipitates were meticulously dried at 80 °C in a vacuum oven until a constant weight was obtained, yielding the PVDF-g-mPEGA product.

The degree of grafting (DG) of PVDF-g-mPEGA is defined as Equation (1):

(1) $$\mathrm{DG}=\frac{W_{1}-W_{0}}{W_{0}}\times 100\%$$

where *W*_1_ denotes the mass of PVDF-g-mPEGA, and *W*_0_ is the mass of pristine PVDF.

### 2.3. FTIR Characterization

The attenuated total reflectance infrared (ATR-IR) spectrum of the pristine PVDF and the grafted polymer was analyzed using a Nicolet Avatar 370 infrared spectrometer (Thermo Nicolet Instrument Corporation, Madison, WI, USA). Spectral scans were conducted over a range of 400 cm^−1^ to 4000 cm^−1^ with a resolution of 4 cm^−1^, and each scan was repeated 32 times to ensure accuracy.

Given the challenges in pulverizing the grafted polymer PVDF-g-mPEGA, the solution membrane method was selected for sample preparation. The procedure involved dissolving the grafted polymer in NMP solvent at a 1:9 ratio, stirring the mixture at 70 °C for 24 h, and then allowing it to stand for defoaming. The homogenized solution was then poured onto a glass plate in a controlled environment with air humidity maintained at 45% ± 5% and a temperature of 24 °C ± 1 °C. The solution was evenly spread using a 50 μm spatula and left to stand in air for approximately 15 s. Subsequently, the glass plate was placed horizontally in water at 20 °C, allowing the membrane to detach automatically. The membrane was then marked and immersed in deionized water, with water changes conducted several times. After 24 h of soaking, the membrane was transferred to a vacuum drying oven for drying, making it ready for subsequent use.

### 2.4. Phase Diagram Drawing

The phase diagram was constructed using cloud point titration. Initially, a series of polymer solutions were prepared with concentrations of 0.2%, 0.4%, 0.6%, and 0.8%, using NMP as the solvent. These solutions were then stirred continuously at a stable temperature of 25 °C. Water was gradually added dropwise until the initially transparent polymer solution turned cloudy. The composition of the solution at the moment of cloudiness, marked by the addition of the last water drop, is defined as the cloud point. Based on the data obtained from cloud point titration, phase diagrams for different solution systems were then plotted.

### 2.5. Rotational Viscosity

The viscosity of polymer solutions with varying compositions was analyzed using an NDJ-79A rotary viscometer (Tryte Technology (H.K.) Limited, Hong Kong, China) as the rotational speed was altered. Initially, the samples were heated and stirred in a solute-to-solvent ratio of 1:9 to achieve a homogeneous solution. After cooling the solution to room temperature, the viscosity measurements were initiated at a rotational speed of 80 r/min. The speed was then incrementally increased by 20 r/min steps, reaching a maximum of 400 r/min.

### 2.6. Membrane Preparation

The membranes were fabricated using the non-solvent-induced phase separation (NIPS) method, as described in our group’s previous research [9]. Initially, PVDF (5.0 g), graft polymer (5.0 g), and PEG20000-enhanced PVDF (4.5 g PVDF with 0.5 g PEG20000) were dissolved in NMP (35.0 g). This mixture was heated and stirred at 70 °C for one week, followed by a 12-h stabilization period to allow bubble release. Subsequently, the prepared solution was spread onto a glass plate using a casting knife to achieve a uniform thickness of 200 μm. The spread solution was then left to evaporate in ambient air for 15 s before being immersed in deionized water. The membranes were carefully stored in water for two days to ensure complete removal of any residual solvent before testing. The water was changed several times during this period.

### 2.7. Water Contact Angle Measurements

Membrane contact angle measurements were performed utilizing an Attension Theta system (KSV Instruments Ltd., Helsinki, Finland). A droplet of 5 μL was deposited from a needle tip onto the membrane surface, and its magnified image was recorded using a digital camera. Static contact angles were then determined from these images using computational software. To guarantee accuracy and consistency, the contact angle values were averaged from three separate locations on each membrane.

### 2.8. SEM Analysis

Membrane morphology was examined using a LEO1530vp scanning electron microscope (Zeiss, Jena, Germany). To obtain the cross-section images, membranes were immersed in liquid nitrogen to freeze them, and then they were cracked in a brittle state. Samples were then mounted on the stage, and a thin gold coating was applied before examination. The scanning was conducted at a voltage of 10 kV and a current of 10 mA. The collected images were processed with the software ImageJ to determine the number of pores per unit area, pore sizes, and pore size distribution.

### 2.9. Filtration Experiment

**Figure 2:**
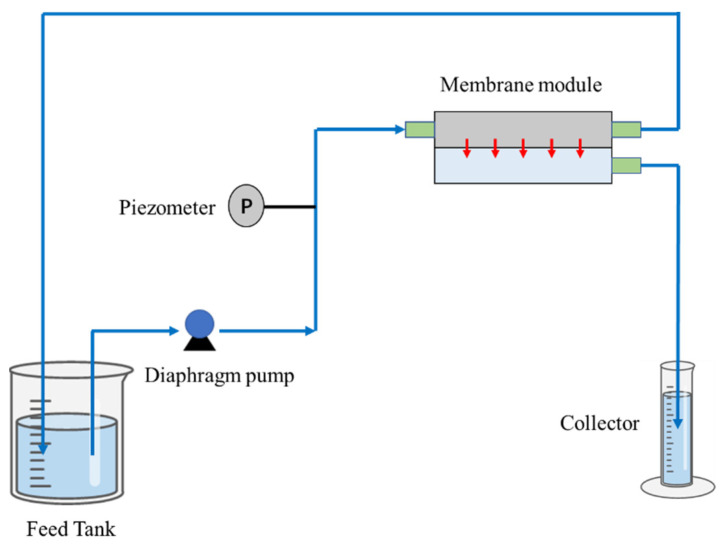
Schematic diagram of the cross-flow device used in the filtration experiments (The control of trans-membrane pressure is regulated through the utilization of a diaphragm pump).

The performance of various membranes was assessed using a custom-built cross-flow filtration apparatus (Figure 2). Circular samples, each with a surface area of 15.9 cm^2^, were prepared and installed in flat cross-flow cells. Membranes were first subjected to a 20-min pre-compression at 0.1 MPa. Three independent trials were performed for each membrane. Flux was calculated by measuring the volume of solution that permeated over time and normalizing it to the membrane area.

The water flux (*F*_0_) was calculated according to Equation (2):

(2) $$F_{0}=\frac{Q}{A\Delta T}$$

where *Q* refers to the volume of the filtered water; *A*, the area of the membrane; and Δ*T*, the time of the filtration.

The preparation of the BSA solution entailed several steps. Initially, a mixture comprising 8 g of NaCl, 1.42 g of Na_2_HPO_4_, 0.2 g of KCl, 0.27 g of KH_2_PO_4_, and 800 mL of deionized water was thoroughly mixed in a volumetric flask. The pH of the resulting solution was adjusted to 7.4 using HCl. The final volume was then brought up to 1 L. Following this, 1 g of BSA was dissolved in the prepared buffer solution, resulting in a feed solution with a BSA concentration of 1 g/L. Samples before and after filtration were carefully collected for subsequent analysis. The determination of BSA concentration in the solution was conducted using a UV-1100 visible-ultraviolet spectrophotometer (Ruili Instruments Ltd., Beijing, China), as outlined in the referenced literature [50].

### 2.10. Anti-Fouling Evaluation

The antifouling properties of the membranes were assessed from two perspectives. The first involved analyzing the recovery ratio of pure water flux post-BSA solution filtration. The second entailed evaluating membrane performance under repeated fouling and cleaning cycles. After a 30-min filtration of the BSA solution, the solution in the cross-flow system was replaced with deionized water. This was followed by a 30-min dedicated cleaning process focused on the membrane surface, culminating in the measurement of water flux recovery.

The flux recovery ratio (*FRR*) is calculated according to Equation (3):

(3) $$FRR=\frac{F_{1}}{F_{0}}\times 100\%$$

where *F*_0_ and *F*_1_ denote the water flux prior to and following fouling, respectively.

## 3. Results and Discussions

### 3.1. Kinetics of the Grafted Polymer

**Figure 3:**
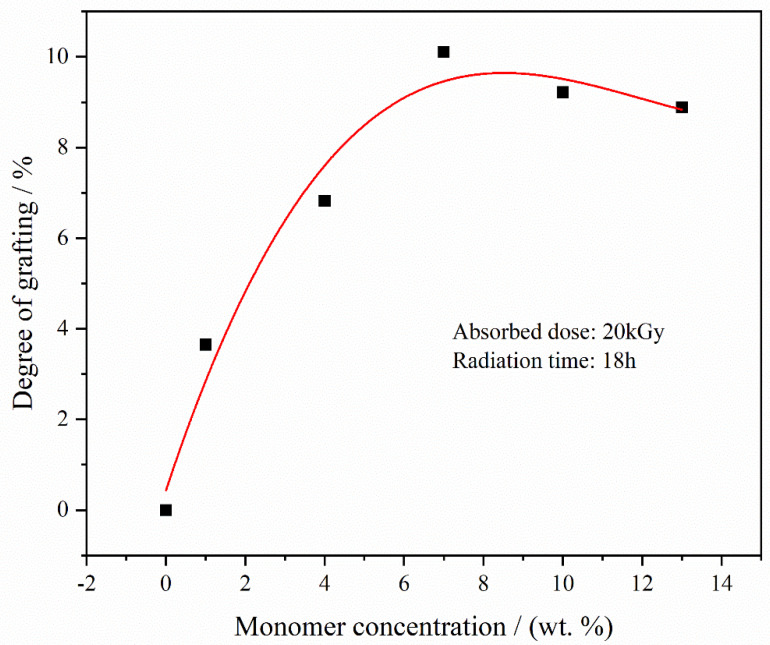
DGs of PVDF-g-mPEGA at different monomer concentration (absorbed dose = 20 kGy; radiation time = 18 h).

The influence of grafting was investigated, considering three variables: Monomer concentration, absorbed radiation dose, and radiation duration. Figure 3 depicts how monomer concentration affects grafting. With a constant absorbed dose of 20 kGy and a radiation time of 18 h, the degree of grafting noticeably increased as the monomer concentration rose from 1% to 7%. Beyond 8% concentration, the grafting level plateaued and then decreased. This pattern is likely due to the enhanced potential for free radical-initiated graft polymerization at higher monomer concentrations, which initially increases grafting. However, at certain absorbed doses, the number of active sites becomes saturated. Consequently, homopolymerization competes with graft copolymerization for these sites. An increase in monomer concentration leads to a higher viscosity in the graft copolymerization solution, affecting monomer solution diffusion. Furthermore, the homopolymerization reaction between monomers can reduce the degree of grafting (DG). Therefore, an increase in monomer concentration does not continuously correlate with an increase in DG.

**Figure 4:**
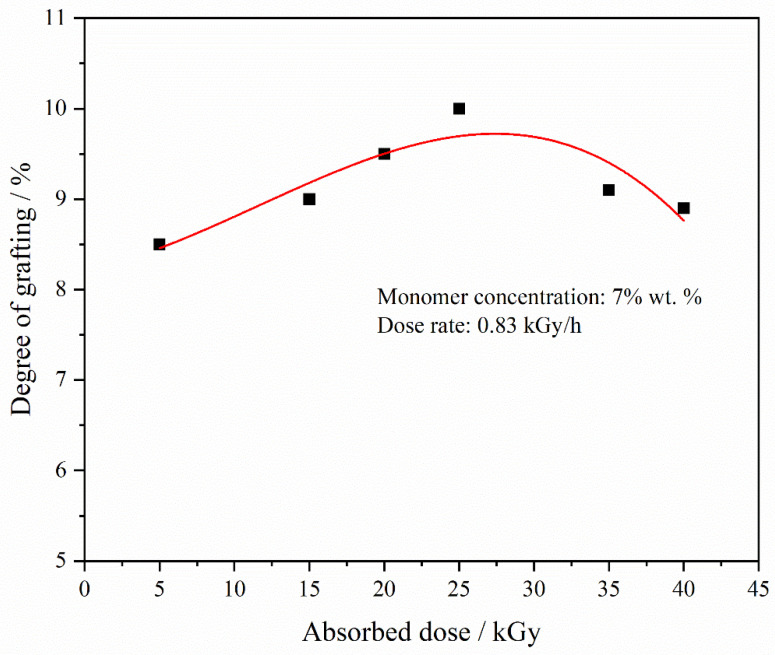
DGs of PVDF-g-mPEGA at different dose rate (monomer concentration = 7 wt.%, dose rate = 0.83 kGy/h).

Figure 4 illustrates the effect of absorbed dose on grafting degree under specified conditions, with a constant monomer concentration of 7% and a radiation dose rate of 0.83 kGy/h. The analysis indicated a moderate impact of the absorbed dose on grafting degree. For absorbed doses ranging from 5 kGy to 40 kGy, the grafting degree of the polymer graft mostly ranged between 8.5% and 10%. It was also noted that up to an absorbed dose of 25 kGy, there was a marginal increase in grafting degree with a rising absorbed dose. Beyond 30 kGy, however, this trend reversed. This shift is attributed to the degradation or cross-linking of PVDF at higher radiation doses [51], resulting in a decrease in free radical active sites available for polymerization, thereby reducing the DG.

**Figure 5:**
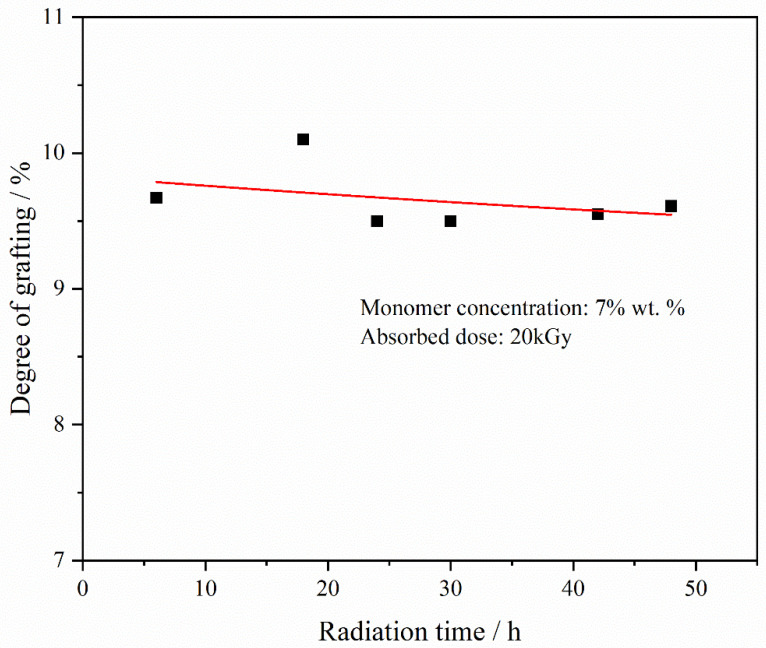
DGs of PVDF-g-mPEGA at different radiation time (monomer concentration = 7 wt.%, absorbed dose = 20 kGy).

Figure 5 illustrates the effect of irradiation time on the degree of grafting. Under fixed monomer concentration and absorbed dose conditions, variations in irradiation time had minimal impact on grafting degree. This outcome significantly deviates from the established behavior of homogeneous radiation grafting in small-molecule monomers [9]. The larger molecular weight of mPEGA, compared to smaller molecular monomers, results in a reduced molar concentration of carbon-carbon double bonds available for free radical combination during irradiation, given an equal mass of monomers. Additionally, homogeneous solution radiation grafting causes molecular chains to extend within the solution, thereby accelerating the diffusion process of the graft reaction. Consequently, the grafting reaction tends to reach saturation rapidly.

### 3.2. FT-IR Spectroscopy of Grafted Copolymers

**Figure 6:**
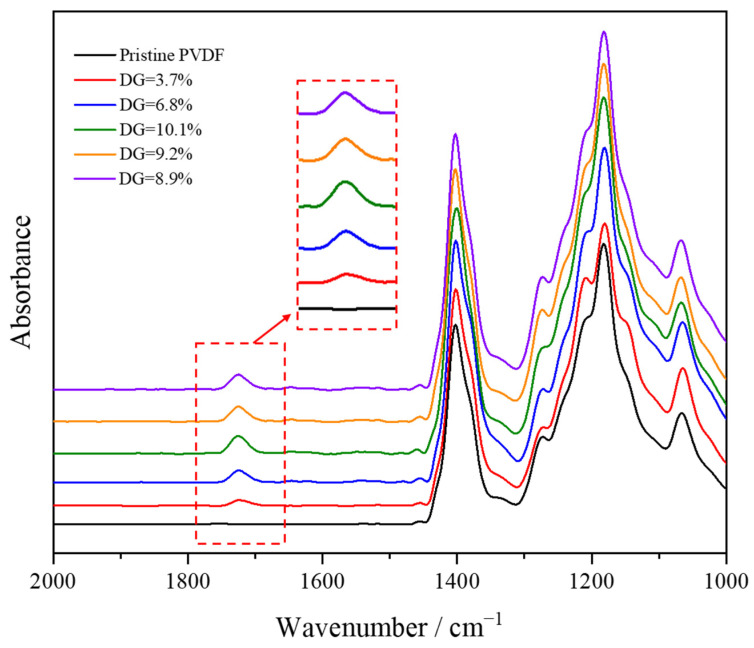
FTIR spectra of the pristine PVDF and PVDF-g-mPEGA copolymers with different DGs.

Figure 6 displays the infrared spectra of pristine PVDF and PVDF-g-mPEGA with different degrees of grafting (DG) after vector normalization. The characteristic C-F absorption peak, around 1072 cm^−1^, was noticeable in both the grafted polymer and the original PVDF, while the CF_2_ vibrational absorption peak appeared at 1182 cm^−1^. Additionally, the deformation vibration absorption peak of CH_2_ was present at 1404 cm^−1^. Notably, the grafted polymer exhibited a new characteristic peak at 1726 cm^−1^, associated with the stretching vibration of the carbonyl group in the mPEGA molecule [52,53]. This peak confirms the successful irradiation grafting of the monomer onto PVDF. Furthermore, a trend was observed where the intensity of the carbonyl peak increased with the degree of grafting.

### 3.3. Ternary Phase Diagram

**Figure 7:**
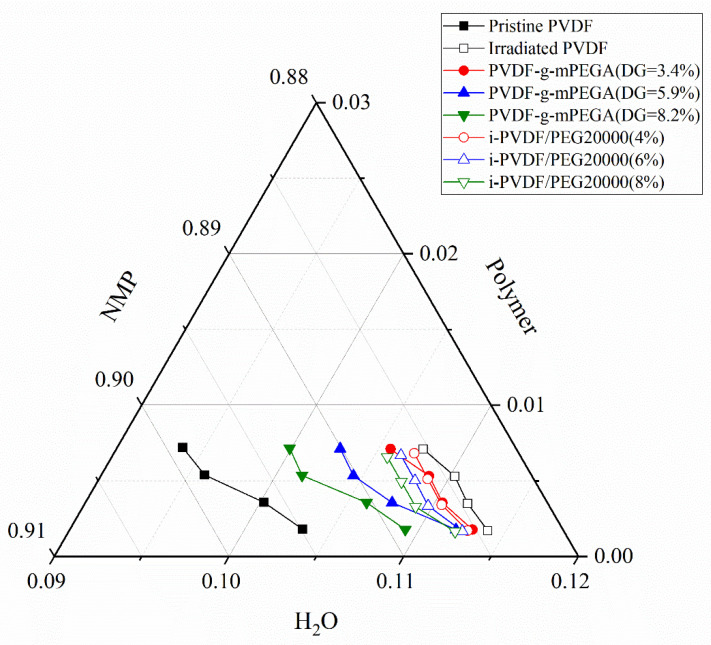
Polymer-NMP-H_2_O ternary phase diagram.

This study examined pristine PVDF, irradiated PVDF, PVDF/PEG20000 blends with varying ratios, and PVDF-g-mPEGA with different DG, all titrated with ultra-pure water using NMP as the solvent. Figure 7 demonstrates that phase separation in the PVDF ternary system was more challenging post-irradiation, necessitating greater amounts of water. The propensity for phase separation increased with higher DG and PEG20000 content. However, it was noted that blending’s effect on the system was considerably less than that of grafting. The enhanced thermodynamic stability post-irradiation could be attributed to PVDF degradation and the presence of smaller molecules. Conversely, increased grafting and blending ratios led to a decrease in thermodynamic stability, likely due to the reduced PVDF content in the mixture. A more significant decline in thermodynamic stability was observed in graft copolymers, possibly due to an increase in graft copolymer molecules. Consequently, during phase separation, the speed of this process in the casting solution of PVDF-g-mPEGA was accelerated with increasing DG. This finding implies that membranes manufactured using the same concentration of the polymer-solvent system, but with higher grafting degrees in PVDF-g-mPEGA, are more prone to exhibit pronounced surface loosening.

### 3.4. Rotational Viscosity Test

**Figure 8:**
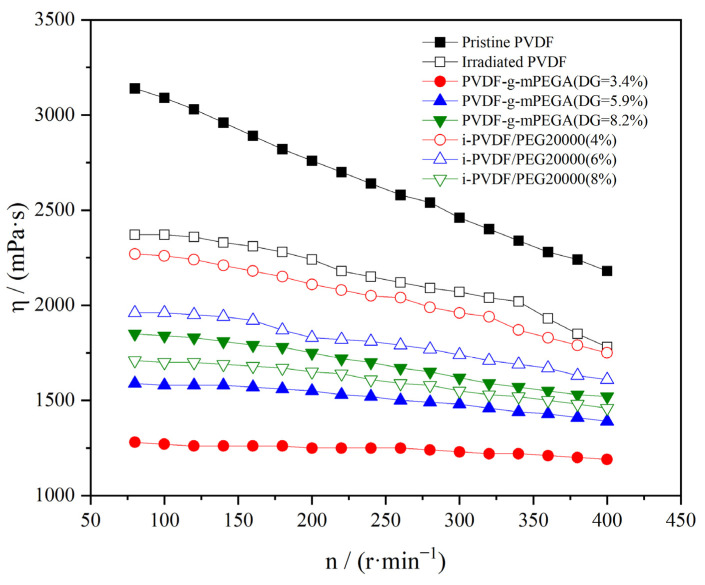
Diagram of the rotating viscosity of different samples as a function of rotational speed.

Figure 8 presents a graph illustrating the variation in rotational viscosity of various samples with changes in rotational speed. As depicted, the rotational viscosities of all samples demonstrated a decline to different extents as the rotational speed increased, categorizing PVDF as a pseudoplastic non-Newtonian fluid [54,55]. Concurrently, it was observed that irradiation reduced the rotational viscosity of PVDF, indicating its degradation. However, the impact of alterations in grafting ratio and blending ratio on rotational viscosity was contrary: Both grafting and blending individually led to a decrease in rotational viscosity. Notably, an increase in the DG resulted in higher rotational viscosity, while an increased blending ratio lowered it. This viscosity reduction can be attributed to the decreased PVDF content in the total composition. In contrast, compared to the mixed system, a rise in DG led to increased viscosity, possibly due to the augmentation of graft content and molecule size. This distinction further confirms that mPEGA was grafted onto PVDF, rather than merely blended.

### 3.5. Water Contact Angle Test

**Figure 9:**
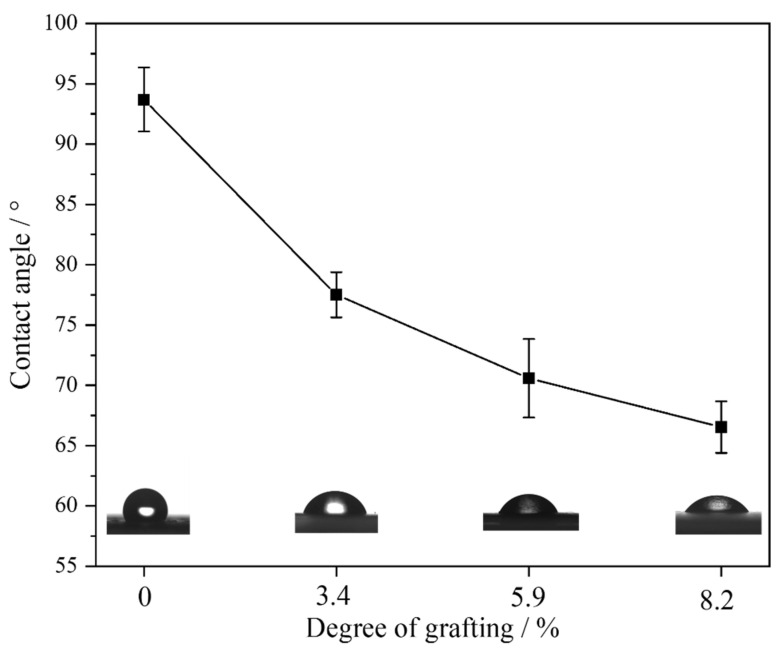
Water contact angle of pristine PVDF and PVDF-g-mPEGA with different DGs. The static water contact angle images corresponding to individual membranes are presented below the line graph.

It is widely recognized that enhancing the hydrophilicity of a membrane typically improves both its flux and antifouling properties [56,57]. Figure 9 demonstrates this principle, showing the contact angle of pristine PVDF prior to grafting as 93.8°, indicative of significant hydrophobicity, consistent with the inherent characteristics of PVDF materials. At a grafting degree of 3.4%, the contact angle decreased to 77.4°. Further, there was a continuous decrease in water contact angle with increasing degrees of grafting, reaching 66.4° at a grafting degree of 8.2%. Thus, it is evident that the grafted PVDF exhibited a substantial improvement in hydrophilicity.

### 3.6. Membrane Morphology Characterization

**Figure 10:**
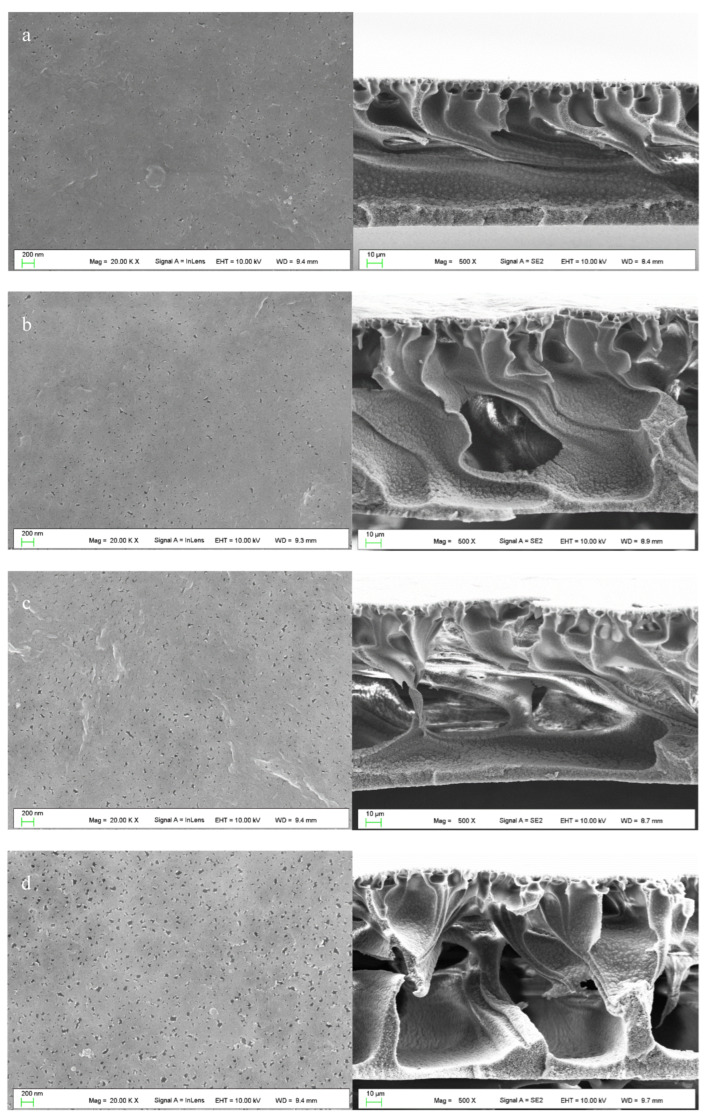
SEM images of the membrane surface (left) and cross section (right): (**a**) pristine PVDF, (**b**) PVDF-g-mPEGA (DG = 3.4%), (**c**) PVDF-g-mPEGA (DG = 5.9%), (**d**) PVDF-g-mPEGA (DG = 8.2%).

**Figure 11:**
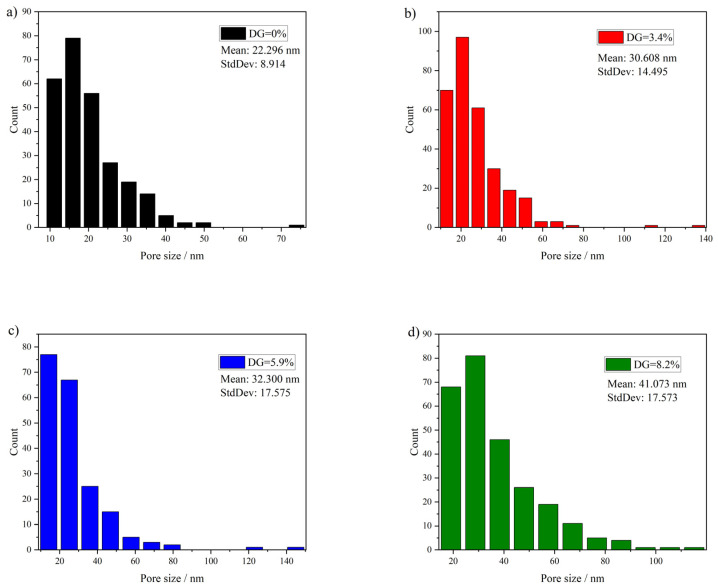
Histogram of the pore size distribution calculated using the surface SEM images. (**a**) Pristine PVDF, (**b**) PVDF-g-mPEGA (DG = 3.4%), (**c**) PVDF-g-mPEGA (DG = 5.9%), (**d**) PVDF-g-mPEGA (DG = 8.2%).

Figure 10 illustrates the surface and cross-sectional structures of the membrane, while Figure 11 displays the pore size distribution histogram derived from surface SEM images calculated by using ImageJ. The findings reveal that all membrane surfaces exhibit improved nanoscale pore structures. The pore size shows an increasing trend with the increase in DG. The cross-sectional SEM image of the PVDF membrane confirmed its asymmetric structure, characteristic of such membranes. The uppermost layer of the PVDF membrane consists of a dense skin layer, whereas the lower surface features a supportive structure. As depicted in Figure 10, grafting did not modify the skin-finger structure of the membrane. However, a comparative analysis of the support layer structures under different membranes revealed that, unlike the pristine PVDF membrane, the PVDF-g-mPEGA graft-modified membrane exhibited large macrovoids structures. These structures were interconnected with the pores, extending throughout the entire membrane. The following explanations are proposed, integrating this study with previous experimental results: On the one hand, the increased thermodynamic stability of the grafted polymer will lead to slow initial demixing; that is, liquid–liquid demixing occurs before the gelation process. This creates a polymer-rich phase and a polymer-poor phase, corresponding to the membrane matrix and pores, respectively, and the tiny macrovoids formed in the skin layer. Subsequently, some of the advancing polymer-lean phases may coalesce and advance in a disordered manner, with tiny macrovoids in the skin layer transforming into larger and irregularly shaped macrovoids at the base of the sublayer [58]. On the other hand, as the grafting rate of the graft polymer increases, the content of the hydrophilic graft segment increases, and the diffusion rate of the solvent becomes faster, resulting in a greater tendency to form macrovoids during the membrane formation process [59].

### 3.7. Membrane Filtration Performance

**Figure 12:**
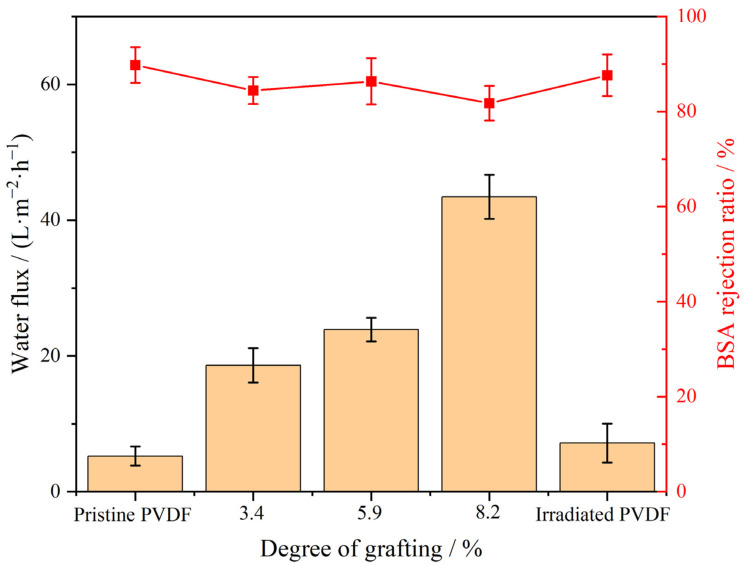
Water flux and BSA rejection of the pristine PVDF, irradiated PVDF, and PVDF-g-mPEGA membranes with different DGs.

Figure 12 presents the water flux and BSA rejection rates of pristine PVDF, irradiated PVDF, and PVDF-g-mPEGA membranes with varying DG. The water flux and BSA rejection rates for the PVDF membrane, both pre- and post-irradiation, showed negligible differences, indicating that the irradiation dose employed had minimal impact on the membrane’s properties. As DG increased from 0 to 3.4%, 5.9%, and 8.2%, the water flux correspondingly rose from 5.2 LMH to 18.6 LMH, 23.9 LMH, and 43.5 LMH. This trend indicates that graft modification of PVDF significantly enhanced its ultrafiltration membrane flux, with a clear positive correlation between higher DG and improved flux. The reasons for this are twofold: Firstly, pure PVDF is known for its strong hydrophobicity, which results in substantial filtration resistance in the PVDF membrane during the water filtration process, necessitating a high driving force for achieving high flux [60,61]. The contact angle test results earlier demonstrated that grafting with the hydrophilic monomer mPEGA significantly increased the hydrophilicity of the modified membrane. This increase in hydrophilicity reduced filtration resistance, leading to a higher flux. The greater the DG, the more pronounced the improvement in hydrophilicity, thereby enhancing the flux. Secondly, the membrane morphology characterization revealed an increase in surface porosity after modification. Additionally, SEM analysis showed that large cavity structures within the support layer of the modified membrane were interconnected with the pores, extending throughout the membrane. This structural arrangement facilitated increased membrane flux. In BSA rejection tests, all types of PVDF membranes consistently achieved BSA rejection ratios over 80%.

### 3.8. Anti-Fouling Evaluation

The assessment of antifouling performance for each membrane typically includes calculating the pure water FRR [62,63]. In this experiment, an in situ measurement technique was employed, integrating online cleaning of the membrane via a cross-flow filtration method. This process entailed alternating the fluid composition, specifically switching between water and a BSA solution.

**Figure 13:**
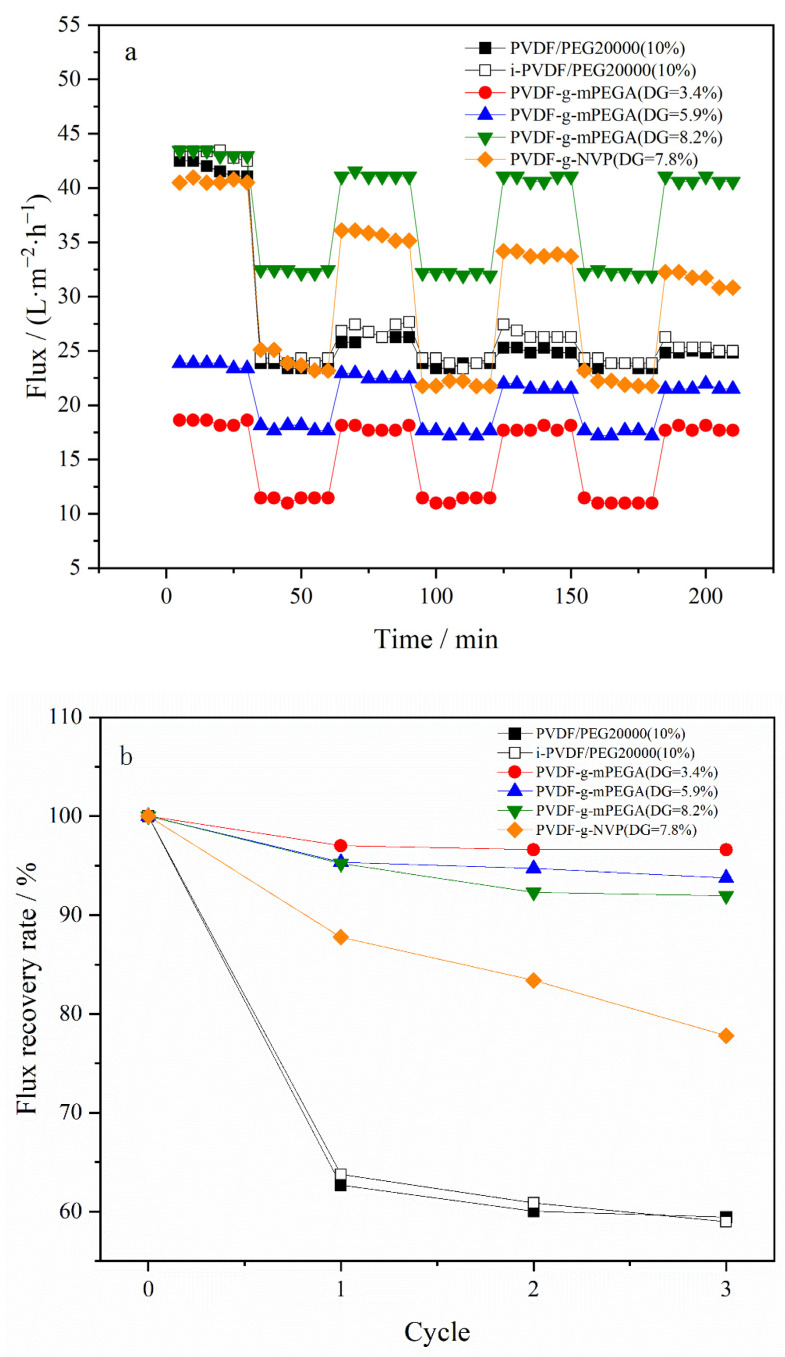
Anti-fouling test of the membranes: (**a**) multiple cycle fouling and cleaning fluxes of different types of PVDF membranes; (**b**) FRRS of different types of PVDF membranes under multiple cycles.

Figure 13a displays the fouling and cleaning flux profiles over multiple cycles for different types of PVDF membranes, whereas Figure 13b presents the corresponding flux recovery rates (FRR) calculated using the data from Figure 13a. PEG20000 was incorporated to enhance the flux of pure PVDF for antifouling evaluation. Notably, membranes grafted with mPEGA demonstrated a significantly higher flux recovery rate compared to PVDF/PEG20000 membranes. The FRR of PVDF-g-mPEGA membranes with various degrees of grafting (DG) remained above 90% and was stable across multiple cycles. These findings highlight the effectiveness of PVDF-g-mPEGA in achieving optimal antifouling performance with minimal grafting, thereby preserving the bulk material properties of the membrane. The flux and FRR of irradiated PVDF/PEG20000 (i-PVDF/PEG20000) membranes were similar to those of PVDF/PEG20000, indicating that irradiation had a negligible impact on the membrane’s formative properties. PVDF-g-NVP, a modified PVDF membrane created using NVP (a commonly used small-molecule monomer suitable for homogeneous radiation) as the graft monomer through the aforementioned methods, showed improved FRR compared to the PVDF/PEG20000 membrane after repeated fouling and cleaning tests. However, its FRR was still considerably lower than that of the PVDF-g-mPEGA membrane. This difference accentuates the unique benefits of mPEGA as a graft monomer in enhancing the antifouling properties of PVDF membranes, especially when compared to NVP.

## 4. Conclusions

In this study, mPEGA, a novel PEGylated monomer, was grafted onto PVDF using homogeneous γ-ray irradiation. Analysis of grafting kinetics revealed that the DG was primarily influenced by monomer concentration, with an increase in DG corresponding to higher concentrations. Thermodynamic analysis of the grafted products indicated that during membrane formation, the phase separation rate of the PVDF-g-mPEGA casting solution accelerated with an increase in DG, thereby enhancing membrane hydrophilicity.

Although irradiation grafting induced some degradation of PVDF, as evidenced by the rotational viscosity test and phase diagram, both the flux and anti-fouling tests demonstrated minimal impact on the membrane-forming properties of PVDF. Moreover, a range of membranes was fabricated using the NIPS method, and SEM analyses confirmed an expansion in membrane pore size commensurate with increasing DGs. The flux and anti-fouling assessments consistently highlighted the superior anti-fouling properties of the PVDF-g-mPEGA membrane compared to the PVDF-g-NVP membrane. Significantly, PVDF-g-mPEGA achieved the highest anti-fouling effect at a very low DG, thus minimizing alterations to the membrane’s bulk material.

From an overall perspective, in addition to better performance, the scalability, processability, and cost of the manufacturing method, as well as the long-term stability of the membrane, all need to be considered [64]. In this study, the anti-fouling cycle test was only conducted for more than 200 min, so the long-term sustainability of the membrane’s performance requires further study. However, the fabrication method of the membrane in this work is simple, making it more scalable and having a low manufacturing cost from an industrial perspective.

## Figures and Tables

**Table 1:** Recipe for synthetic PVDF-g-mPEGA.

No.	PVDF/g	mPEGA/g	NMP/g	Total/g
**1**	10	1	89	100
**2**	10	4	86	100
**3**	10	7	83	100
**4**	10	10	80	100
**5**	10	13	77	100

## Data Availability

Data are contained within the article.
